# From blender to farm: Transforming controlled environment agriculture with synthetic data and SwinUNet for precision crop monitoring

**DOI:** 10.1371/journal.pone.0322189

**Published:** 2025-04-24

**Authors:** Kimia Aghamohammadesmaeilketabforoosh, Joshua Parfitt, Soodeh Nikan, Joshua M. Pearce

**Affiliations:** 1 Department of Electrical & Computer Engineering, Western University, London, Ontario, Canada; 2 College of Engineering, Pennsylvania State University, University Park, Pennsylvania, United States of America; 3 Ivey Business School, Western University, London, Ontario, Canada; The University of Tokyo: Tokyo Daigaku, JAPAN

## Abstract

The aim of this study was to train a Vision Transformer (ViT) model for semantic segmentation to differentiate between ripe and unripe strawberries using synthetic data to avoid challenges with conventional data collection methods. The solution used Blender to generate synthetic strawberry images along with their corresponding masks for precise segmentation. Subsequently, the synthetic images were used to train and evaluate the SwinUNet as a segmentation method, and Deep Domain Confusion was utilized for domain adaptation. The trained model was then tested on real images from the Strawberry Digital Images dataset. The performance on the real data achieved a Dice Similarity Coefficient of 94.8% for ripe strawberries and 94% for unripe strawberries, highlighting its effectiveness for applications such as fruit ripeness detection. Additionally, the results show that increasing the volume and diversity of the training data can significantly enhance the segmentation accuracy of each class. This approach demonstrates how synthetic datasets can be employed as a cost-effective and efficient solution for overcoming data scarcity in agricultural applications.

## 1. Introduction

Accurate identification of fruit maturity plays a crucial role in agriculture, influencing the optimization of harvesting processes, reducing waste, and improving crop quality. Traditionally, fruit ripeness has been assessed through manual inspection, but these methods are labor-intensive, time-consuming, and prone to human error. Recent advancements in deep learning for agricultural applications have focused on lightweight object detection models and robotic harvesting techniques. For instance, a lightweight improved YOLOv5s model has been successfully deployed for detecting pitaya fruits in both daytime and nighttime conditions using enhanced light-supplement environments [1]. Similarly, dynamic visual servo control methods have been employed for autonomous fruit harvesting, enabling continuous operation of robotic harvesters in complex orchard environments [2]. However, while these models excel at object-level detection, they do not perform fine-grained pixel-wise segmentation required for distinguishing individual fruit components and maturity stages. To address these challenges, the application of computer vision techniques, especially deep learning models, has emerged as a promising solution for automating the identification of ripe and unripe fruits with improved efficiency and accuracy.

Recent advancements in deep learning, particularly with Vision Transformer (ViT) models, have demonstrated significant potential in fruit classification and disease detection tasks. ViT-based methods have shown remarkable performance in detecting strawberries and assessing their ripeness. Zheng et al. [3] explored ViT models for strawberry quality classification, integrating a Support Vector Machine (SVM) and achieving an impressive accuracy of 98.1%. Further studies applied ViT models enhanced with transfer learning for strawberry disease detection, classifying diseases across various categories and achieving an F1-score of 0.927 on the Strawberry Disease Detection dataset [4]. Additionally, LS-YOLOv8s, a model incorporating the LW-Swin Transformer module, significantly improved ripeness detection with 94.4% precision [5]. In a recent study, the ViT model was fine-tuned on augmented strawberry images, achieving an accuracy of 98.4% and a precision of nearly 99% for disease classification and ripeness detection [6]. The overall success of these models demonstrates their effectiveness in agricultural applications, achieving high accuracy while maintaining relatively low computational costs. Standard ViT models are limited, however, by their inability to perform semantic pixel-level classification, a requirement for more complex tasks like semantic segmentation. To overcome this limitation, advanced architectures such as SETR and Swin Transformer have been developed for segmentation tasks [7,8]. Semantic segmentation plays a crucial role in agriculture, particularly in precision farming, by enabling robots to detect and classify crops, weeds, and other elements with high precision [9]. Conventional methods, such as CNN-based models, have been widely used for segmentation tasks, distinguishing between crops and background solely using RGB data. Recent studies have demonstrated the effectiveness of ViTs in precision agriculture tasks, including weed detection [10,11], aerial object counting [12], and multimodal segmentation [13]. The role of AI-based environmental modeling in improving synthetic data approaches for agriculture has been emphasized in recent work by Mampitiya et al. [14], reinforcing the significance of automated crop monitoring methods in controlled and open-field conditions. While studies such as NWPU-MOC [12] focus on object counting rather than segmentation, other works, such as Swin-Unet for weed identification [10] and multiclass weed segmentation [11], highlight the adaptability of ViTs for agricultural analysis. The approach used in this study builds upon these insights by applying transformer-based segmentation to fruit maturity assessment.

In response to the limitations of ViT models, hybrid architectures like SwinUNet have been developed. SwinUNet [15] combines the strengths of Swin Transformers, which employ shifted windows for self-attention, with the proven U-Net structure [16]. SwinUNet enhances performance by effectively managing multi-scale image features, maintaining detailed context, and delivering fine segmentation accuracy. While SwinUNet is commonly used in medical imaging, it has recently been applied to agricultural tasks such as fruit ripeness detection and crop segmentation [5]. The model has demonstrated significant improvements over traditional CNN-based models, especially in tasks requiring high precision and computational efficiency [5,15]. Its flexibility in adapting to various image sizes and types further underscores its potential as a versatile tool across various domains, including agriculture and medical imaging.

A critical requirement for training ViT models is the availability of large datasets [17]. Dosovitskiy et al. [18] demonstrated that the performance of ViT models improves with the increasing size of the training data. However, obtaining large, annotated datasets in agriculture can be challenging due to seasonal availability and the need for time-consuming manual data collection. To address these issues, two common strategies are employed: data augmentation and the use of synthetic data.

Data augmentation (DA) involves artificially expanding training datasets by applying transformations such as flipping, rotating, and scaling to existing images, simulating various noise factors encountered in real-world conditions. Meanwhile, synthetic data generation has gained traction as a scalable and cost-effective alternative [7].

Blender is a free, open-source 3D graphics software that encompasses the entirety of the 3D process, including modeling, animating, rendering, and compositing [19]. It offers several key advantages that make it ideal for generating synthetic datasets. First, Blender is equipped with a physics-based rendering engine [20], making it capable of producing high-quality, photorealistic images. A particular advantage of using Blender is its ability to precisely control the appearance of surfaces and materials through its shader nodes system [21]. An extensive library of textures and backgrounds is also available, enabling a wide variety of realistic objects and environments to be created. Applying these resources can enhance the diversity and realism of synthetic datasets, making them more representative of the real world. The introduction of a node-based procedural workflow in version 2.92 has also significantly enhanced Blender’s capabilities [22]. This feature facilitates the creation and manipulation of complex geometries without the need for manual modeling, allowing for a high degree of flexibility and control over the object. It also enables the randomization of an object’s geometry within each frame, allowing for numerous object variations to be incorporated into a single scene animation. Additionally, Blender supports scripting and automation [23], helping to address issues related to data scarcity and imbalance by providing a scalable and cost-effective solution for generating large volumes of high-quality training data tailored to specific needs.

Previous research has demonstrated Blender’s effectiveness in generating synthetic datasets for various computer vision applications across different domains [24,25]. For example, in the realm of additive manufacturing, Blender has been employed to generate comprehensive datasets for semantic segmentation of 3D-printed parts, improving real-time failure analysis systems by accurately detecting various structural elements [26]. In industrial applications, Blender has been used to create synthetic images for steel defect recognition, leading to improved performance in classifying and segmenting defects on steel slabs [27]. Blender has also been instrumental in developing a quality inspection system for scaffolding, combining synthetic and real datasets to train models for assessing structural safety [28]. In agriculture, Blender has been used to develop synthetic datasets for crop size estimation, effectively addressing challenges such as occlusions and perspective distortions [29]. It has also enabled the creation of realistic datasets for object detection in sweet pepper cultivation through procedural generation, enhancing the training of deep learning models for both object detection and semantic segmentation [30].

A common challenge associated with synthetic datasets is domain disparity [31]. Models trained on synthetic data may struggle to generalize effectively to real-world images due to differences in data distributions between the source (synthetic) and target (real) domains. Domain adaptation techniques offer a solution to this issue by aligning the distributions of source and target domains, enhancing model performance without requiring extensive retraining on new data [32]. In this study, the source domain consists of synthetic images generated using Blender, and the target domain comprises real images from the StrawDI dataset [33].

One of the most effective domain adaptation techniques is Deep Domain Confusion (DDC) [32], which addresses domain shift by integrating a domain confusion loss into the training process. This approach encourages the model to learn domain-invariant features, improving its generalizability across different datasets [17]. DDC has been successfully applied in various fields, including medical imaging, where models trained on one set of MRI scans were effectively adapted to new datasets from different MRI machines, significantly improving performance [34].

This study aims to train a ViT-based segmentation model to differentiate between ripe and unripe strawberries using synthetic data generated with Blender. To overcome the limitations of conventional data collection methods, Blender was used to create a diverse set of synthetic images and corresponding masks, providing an effective training dataset for the model. SwinUNet was employed to do the segmentation through transfer learning, while Deep Domain Confusion was used to address domain disparity and improve model performance on real images. The trained model was subsequently tested on real-world data from the Strawberry Digital Images (StrawDI) dataset [33], demonstrating the potential of synthetic data and domain adaptation techniques in improving agricultural practices.

## 2. Methods

### 2.1 Blender model and synthetic data generation

**Fig 1:**
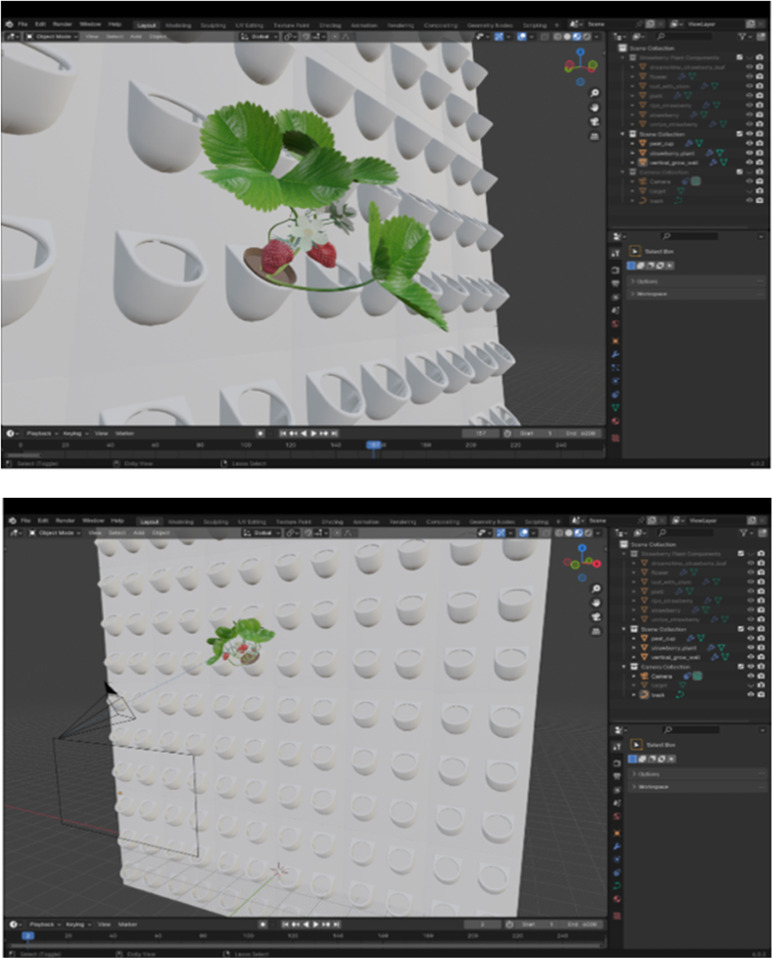
Blender scene showcasing strawberry plant (top) alongside a vertical grow wall and a camera mounted on a track (bottom).

In this study, Blender version 4.0.2 was employed to generate the synthetic dataset of strawberry plants for computer vision training. The scene (shown in Fig 1) comprised a vertical grow wall, peat cups with soil for a strawberry plant, a single strawberry plant, a track for the camera, and the camera itself. It was intended to closely resemble the growing conditions of strawberries planted in an indoor vertical grow wall located in the agrivoltaic agrotunnel at the Western Innovation for Renewable Energy Deployment in London, ON, Canada [35]. Lighting within the scene was achieved through the ambient illumination provided by the default forest environment texture, which mimicked natural light conditions.

The strawberry plant model used in this study was developed using Blender’s geometry nodes [22], a procedural modeling tool that allows for the creation of complex, modifiable plant structures. The plant architecture was designed by dividing the model into constituent parts: stem, leaf, fruit, calyx, and flower. These elements were modeled individually using geometry nodes to generate their shapes and spatial distributions. Afterwards, they were combined using Blender’s Join Geometry node [36].

For the stems, a set of curves with variable noise parameters was used to create randomized shapes, simulating natural stem growth. The Instance on Points node [37] was applied to attach leaves and fruits along the stems. Leaves were modeled as geometric clusters, with each cluster containing multiple leaf shapes to add variability. Similarly, fruits were modeled as a series of stages from unripe to ripe, using a base geometric shape modified by noise and texture gradients to mimic the natural appearance of strawberries at different stages of growth.

Flowers were created using Bézier curves for the petal and calyx structures, which were further adjusted using rotation, scaling, and bending modifiers to ensure a realistic appearance. Textures and materials were applied to all elements to enhance realism, with each material consisting of gradient colors, noise textures, and bidirectional scattering distribution functions (BSDF) for translucency and surface roughness.

The model allowed for procedural variations in the number, size, and orientation of stems, leaves, fruits, and flowers, enabling the creation of multiple plant instances with individual characteristics. To further enhance the model’s variability, the random node [38] was incorporated into the node network, enabling the randomization of parameters such as leaf size and orientation, stem curvature, and strawberry color. The randomization was carefully constrained within realistic limits to avoid non-realistic appearances, ensuring that each rendered frame was unique. While this occasionally resulted in strawberries being covered by leaves, and object intersection, such as overlapping leaves, was a challenge, it generally did not detract from the realism and was often negligible.

Several improvements were also made to the original leaf model. The shape of the leaf was refined to more closely align with a strawberry plant’s true leaf morphology by utilizing Blender’s knife tool [39] to cut out the shape from an imported image-as-plane, based on a reference image [40]. A shader nodes setup [21] was created that allowed for variation in leaf color between lighter and darker green. Additionally, the ability to randomly vary the leaf’s curl was introduced. Finally, the Principled BSDF node [41] was used to increase the roughness of the leaf’s surface, further improving its realism.

Object instancing was used to manage the high number of individual components efficiently. Each instance was subjected to the same randomization parameters, maintaining consistency across different plant parts while ensuring variability between frames. The camera also was set on a track and animated to move along it, changing angles and perspectives in each frame. This method allowed for the creation of highly realistic yet diverse synthetic datasets. Each image contained both ripe and unripe strawberries and was paired with a corresponding mask.

**Fig 2:**
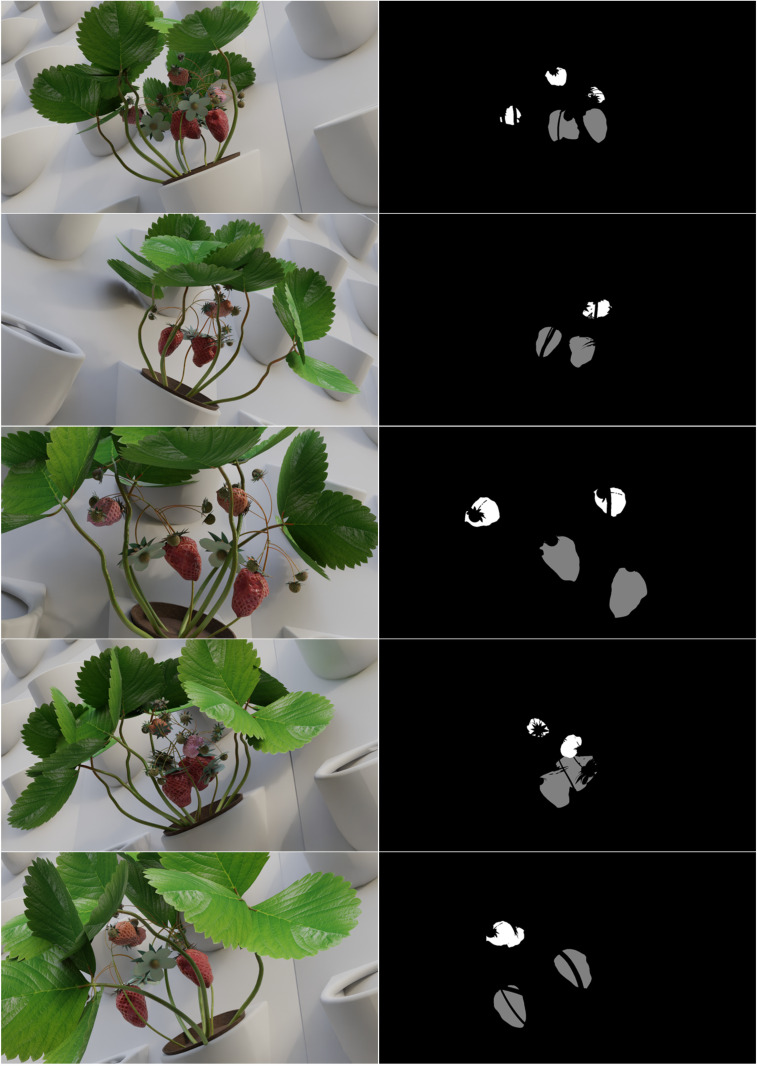
Examples of synthetic images featuring both ripe and unripe strawberries, along with their corresponding masks.

Blender’s compositing tool [42] was used to generate segmentation masks by assigning unique grayscale values to different elements within the image: 0 for the background (black), which included the wall and leaves; 128 for ripe strawberries (gray); and 255 for unripe strawberries (white). Fig 2 displays representative examples of synthetic images featuring both ripe and unripe strawberries, along with their corresponding masks. The cycles rendering engine [20] was used to render the images with the number of samples set to 256. Initially, images were rendered using a CPU, which was later switched to a GPU to decrease rendering time.

### 2.2 Dataset preparation

After generating the synthetic images using Blender, the dataset was then divided into training and validation subsets. The validation set is a subset of the generated synthetic data used during the training process to evaluate the performance of the SwinUNet model on unseen data. To evaluate the model’s real-world performance, a collection of images from the StrawDI dataset [33] was used for testing. However, because these real-world images lacked predefined masks, pre-annotation was required.

**Fig 3:**
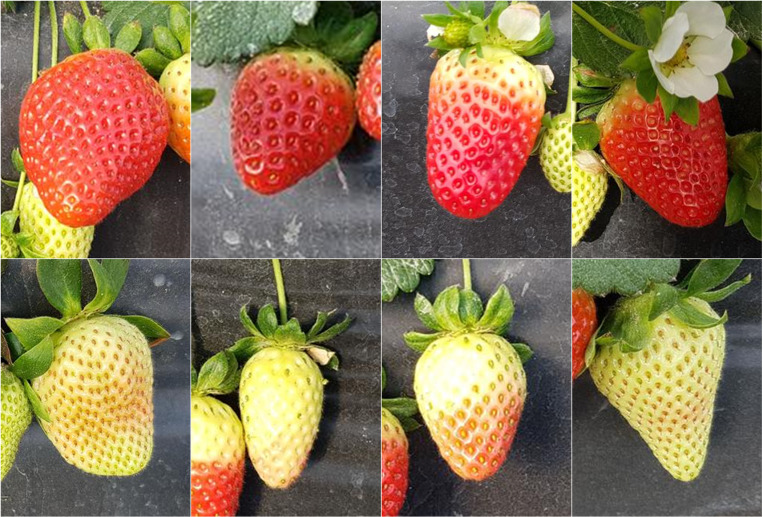
Examples of real test images featuring both ripe (top) and unripe (bottom) strawberries.

**Fig 4:**
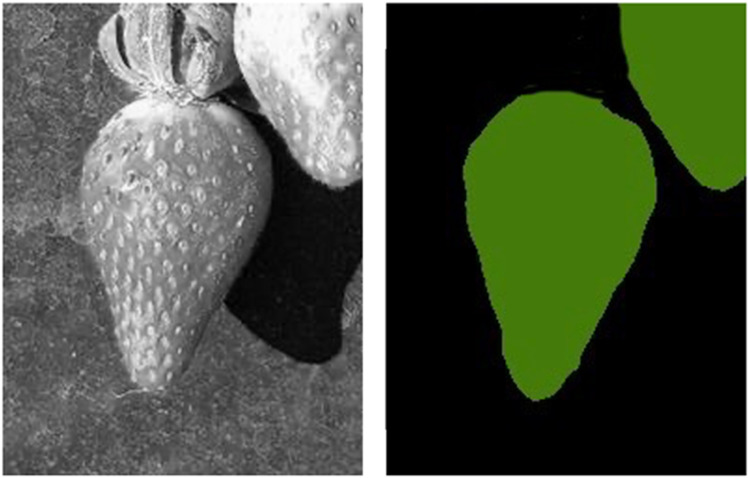
Example of grayscaled real-world image (left) alongside corresponding segmentation mask (right).

For pre-annotation, the real-world images were first converted to grayscale. The masking process was performed using Roboflow [43], a widely-used computer vision tool that facilitates image annotation, dataset creation, and augmentation. The annotated images were used to create segmentation masks for subsequent model testing. Fig 3 presents representative examples of the real test images, and Fig 4 shows a sample grayscaled image alongside its corresponding segmentation mask.

### 2.3 Deep domain confusion

To improve model generalizability across synthetic and real images, Deep Domain Confusion (DDC) was implemented. DDC addresses the problem of domain shift, which occurs when a model trained on one domain (in this case, synthetic data) performs poorly on another (real-world data). By incorporating a domain confusion loss into the training process, DDC encourages the model to learn domain-invariant features. Specifically, a joint loss function is optimized, balancing traditional prediction loss (e.g., cross-entropy) with the domain confusion loss. This approach enhances the generalizability of deep learning models by promoting the extraction of features that are less sensitive to the differences between the source and target domains, thereby improving performance on real-world images. While adversarial domain adaptation techniques such as Domain Adversarial Neural Networks (DANN) introduce a discriminator to align source and target feature distributions, they often suffer from training instability and mode collapse. In contrast, Deep Domain Confusion (DDC) enforces feature alignment directly through domain confusion loss, making it computationally more efficient and robust for small-to-medium agricultural datasets.

### 2.4 SwinUNet

In this study, SwinUNet model was used for image segmentation. SwinUNet [8] is a novel image segmentation model that integrates the hierarchical vision transformer backbone Swin Transformer with the U-Net architecture. Unlike conventional CNN-based segmentation models, Swin Transformer introduces a hierarchical feature representation, leveraging shifted window self-attention mechanisms to efficiently capture both local and global spatial dependencies. This architecture improves upon standard convolutional methods, which are limited by their small receptive fields and lack of long-range feature capture. Compared to U-Net and DeepLabV3+, SwinUNet retains the low computational complexity of CNNs while significantly enhancing segmentation precision through its attention-based mechanisms. Moreover, it has the ability to capture long-range dependencies using a self-attention mechanism. This makes SwinUNet particularly well-suited for tasks involving fine object boundaries, occlusions, and heterogeneous textures, such as fruit ripeness segmentation in complex agricultural scenes. While CNNs perform well in general segmentation tasks, they struggle with occlusions and variable lighting conditions, which are critical challenges in controlled-environment agriculture. By leveraging hierarchical feature representations, SwinUNet provides superior segmentation accuracy in highly occluded fruit environments.

**Fig 5:**
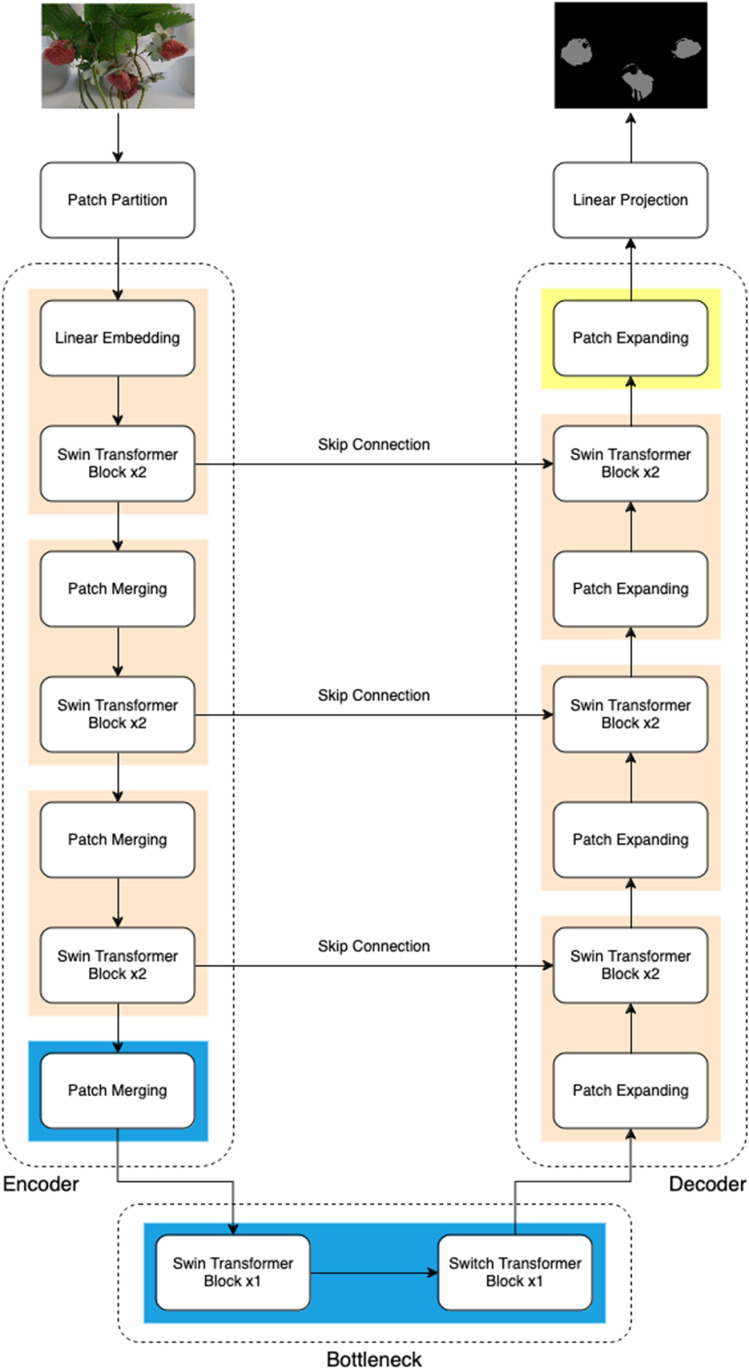
SwinUNet architecture, composed of encoder, decoder, bottleneck, and skip connections.

As shown in Fig 5, adapted from Cao et al. [15], the encoder consists of Swin Transformer blocks, which use shifted windows to efficiently model long-range dependencies in images. The decoder mirrors the encoder, enabling multi-scale feature fusion through skip connections. Each stage of the encoder and decoder includes patch merging (downsampling) or patch expanding (upsampling) layers. The SwinUNet in this study adopts multi-head self-attention and multi-layer perceptron (MLP) layers in its Swin blocks, with ReLU as the activation function. The patch size is 4×4 pixels, and kernel sizes are determined by the Swin Transformer’s window size. For training, the model leverages a combination of cross-entropy loss and Dice loss to enhance pixel-wise classification and account for class imbalance. This model is well-suited for handling the complexities of strawberry ripeness detection task due to its ability to capture multi-scale features and fine details.

### 2.5 Model specifications

The computational resources for this project were provided by the Digital Research Alliance of Canada, which supplied an NVIDIA A100 GPU, a high-performance processor optimized for AI workloads, to accelerate the model training process. The detailed specifications of the model, including hyperparameters and training configurations, are summarized in Table 1.

**Table 1:** Model parameters and specifications.

Parameters	Value
Programming language	Python 3
Architecture	SwinUNet
Domain Adaptation Method	Deep Domain Confusion
Evaluation Metric	Dice Similarity Coefficient
Loss Function	Weighted Cross Entropy Loss
Source Domain	Blender Generated Images
Target Domain	Images from StrawDI Dataset
Computing Power	Digital Alliance of Canada (A100 GPU)
Epochs	400

All code for this study was written in Python and has been made publicly available on the Open Science Framework (OSF) [44] and based on [45].

## 3. Results

This study utilized a Blender-generated synthetic dataset containing 10,000 images, each containing both ripe and unripe strawberries and paired with a corresponding mask. It took approximately 28 hours to generate 10,000 images with their corresponding masks using a GPU.

The trained model was tested on real images from the StrawDI dataset [33], captured using Samsung Galaxy S7 Edge smartphone. By evaluating the model on unseen real-world data, the model’s ability to generalize to non-synthetic environments was thoroughly assessed.

To evaluate segmentation accuracy, we employed the Dice Similarity Coefficient (DSC) [46], a widely used metric in segmentation tasks. The DSC is calculated as 2× | X∩Y | / | X | + | Y | and measures the overlap between the predicted and actual segmentations, making it particularly suited for pixel-wise classification tasks. By comparing the ground truth samples with the predicted segmentations, the DSC provides a measure of accuracy, with values ranging from 0 to 1, with 1 representing a perfect match between the ground truth and segmented results.

The model demonstrated impressive performance on the evaluation set, achieving a DSC of 98.4% for ripe strawberries and 98.1% for unripe strawberries. Notably, the DSC values for the real image test set were slightly lower, with 94.8% for ripe and 94% for unripe strawberries. The observed drop in DSC values from 98.4% (synthetic) to 94.8% (real data) for ripe strawberries and 98.1% to 94% for unripe strawberries can be attributed to key challenges such as occlusions, lighting variations, and synthetic artifacts. These factors introduce domain discrepancies that affect generalization performance. This dataset was intentionally designed to include different occlusion levels, varied lighting conditions, and multiple camera angles to simulate real-world conditions as closely as possible. This provides an implicit sensitivity analysis, demonstrating the impact of these variations on segmentation accuracy. Table 2 displays the results for 10000 training images with each image containing both ripe and unripe strawberries. The model’s high segmentation accuracy on real-world images (DSC of 94.8% and 94% for ripe and unripe strawberries, respectively) demonstrates its robustness to environmental variations, including occlusions, diverse lighting conditions, and multiple camera angles. These results suggest that synthetic-to-real transfer was successful, despite minor domain gaps. Future work will focus on evaluating segmentation performance under extreme lighting variations and real-world farm environments.

**Table 2:** DSC for 10,000 images on validation and test datasets.

	DSC (%) - Validation	DSC (%) - Test
Ripe	98.4	94.8
Unripe	98.1	94
Background	98.7	95.5

To evaluate the DSC scores for different amounts of synthetic training data, we compared the results for 10000 images with those obtained from datasets ranging from 200 to 9000 images. Each trained model was then tested on real images to assess its performance, as shown in Table 3. When the model was trained with only 200 images per class, it achieved a DSC of 58% for ripe strawberries and 56.4% for unripe strawberries on the test set. However, as the training set was gradually expanded to include 1,000, 4,000, 5,000, 6,000, 7,000, 8,000, and finally 9,000 images per class, the DSC improved significantly, reaching 94% for ripe strawberries and 93.7% for unripe strawberries. The substantial improvement that resulted from increasing the amount of training data suggests that while domain disparity remains a challenge, the model’s performance benefits significantly from larger and more diverse training datasets. It is also noteworthy that the DSC scores for ripe strawberries were consistently higher than those for unripe strawberries.

**Table 3:** DSC improvement as number of training images increases.

No. of Images	DSC (Ripe)	DSC (Unripe)
200	58	56.4
1000	78.12	77.29
4000	81.08	79.53
5000	84.9	80.66
6000	89.35	84.47
7000	90.4	89.97
8000	91.08	91
9000	93.7	92.82

Our study demonstrates that Swin-Unet is well-suited for fruit segmentation in controlled-environment agriculture, similar to its application in weed segmentation [10,11]. Unlike multiclass weed identification models, which segment crops from unwanted plants, our work focuses on maturity assessment, requiring fine-grained intra-class segmentation rather than inter-class separation. Additionally, future studies could explore integrating multimodal fusion techniques, as seen in U3M [13], to enhance segmentation accuracy through depth or multispectral data fusion.

## 4. Discussion

The results of this study highlight the effectiveness of the SwinUNet architecture combined with deep domain confusion (DDC) techniques for segmenting ripe and unripe strawberries from synthetic training data. To contextualize these results, this approach can be compared with existing state-of-the-art segmentation models commonly used in agricultural applications. Traditional U-Net-based models have demonstrated high segmentation accuracy in biomedical imaging, but they often struggle with occlusions and lighting variations in agricultural environments. Meanwhile, YOLO-based models such as LS-YOLOv8s [5] are well-suited for real-time object detection but lack fine-grained pixel-wise segmentation capabilities. This study demonstrates that SwinUNet outperforms CNN-based architectures by leveraging transformer-based global attention mechanisms, leading to improved segmentation accuracy in occluded or cluttered agricultural scenes.

Several recent studies have applied deep learning models for agricultural segmentation. U-Net-based architectures, while effective in biomedical applications, often exhibit reduced accuracy in agricultural occlusions [9]. YOLO-based models (e.g., LS-YOLOv8s) achieve real-time detection but lack fine-grained pixel-level segmentation [5]. In comparison, the SwinUNet model used here achieved DSC scores of 98.4% (synthetic) and 94.8% (real) for ripe strawberries, which is competitive with state-of-the-art segmentation models in precision agriculture. Future studies can expand upon these findings by benchmarking SwinUNet against additional transformer-based segmentation methods.

While the dataset used in this study was designed to include occlusions, variable lighting conditions, and multiple camera perspectives, it should be noted that a formal quantitative evaluation of these factors was not explicitly conducted and is left for future work. However, during validation, the model achieved Dice Similarity Coefficient (DSC) values exceeding 98%, demonstrating robust performance, and when tested on real images, the DSC scores dropped slightly, with 94.8% for ripe strawberries and 94% for unripe strawberries. The model’s ability to handle occlusions is attributed to its self-attention mechanism, which enables effective segmentation despite partial fruit coverage. Future work will evaluate segmentation under extreme occlusions and more complex lighting variations. Additionally, advancements in spatial mapping techniques for crop monitoring, as demonstrated by Makumbura et al. [47], further support the need for geospatial analysis tools in precision agriculture. Integrating these methods with vision-based segmentation models could enhance real-time field monitoring applications. While these are still strong results, they reflect the persistent challenge of domain adaptation, as the model’s performance on real-world data lags behind that on synthetic images. Despite the benefits of synthetic data, domain gaps between Blender-generated images and real-world data introduce potential limitations. Minor artifacts, such as inconsistent lighting, unrealistic fruit textures, and overlapping leaf structures, could impact model generalization. Additionally, the synthetic dataset does not fully capture natural variations in environmental factors, such as shadows, varying occlusions, and reflections commonly present in real-world settings. Future work should address these limitations by incorporating physically-based rendering (PBR) techniques and generative adversarial networks (GANs) for synthetic data refinement. The importance of dataset diversity in agricultural AI models has been further highlighted by Kularathne et al. [48], demonstrating how expanded training variability leads to improved generalization performance. This aligns with the results here, where increasing dataset size significantly enhanced segmentation accuracy.

Despite employing DDC to reduce the disparity between synthetic training and real-world test images, it cannot fully bridge the domain gap. The challenges of synthetic-to-real adaptation have been extensively discussed by Fuladipanah et al. [49], emphasizing the need for advanced domain adaptation techniques in precision agriculture. Future work could explore hybrid approaches that combine DDC with adversarial domain adaptation to further improve real-world segmentation performance. DDC helps align features between source and target domains, improving generalization, but cannot eliminate differences in texture, lighting, and other visual factors inherent in real images. This limitation suggests that, while DDC techniques enhance performance, more advanced or complementary domain adaptation strategies are necessary to close the gap and further improve segmentation accuracy on real-world data.

The relationship between training dataset size and segmentation performance was an important aspect of this study. With only 200 images per class, the model performed poorly, achieving DSC values of 58% for ripe and 56.4% for unripe strawberries. However, as the training dataset grew to 9,000 images per class, the DSC scores improved significantly, reaching over 90% for unripe strawberries when tested on real images. This highlights the importance of dataset size in model performance, particularly when dealing with domain disparity.

This trend highlights the importance of sufficient data in improving model performance, especially when dealing with domain disparity between synthetic training data and real test images. The use of DDC techniques helped bridge this gap to some extent, yet the model still benefited greatly from the increased diversity and quantity of synthetic training data. These results suggest that domain adaptation techniques like DDC can be effectively combined with large-scale synthetic datasets to mitigate the challenges of limited real-world annotated data in agricultural settings.

The DSC scores for ripe strawberries consistently exceeded those for unripe strawberries across all training set sizes. This difference may be due to the more distinct visual features of ripe strawberries, such as color, texture, and shape, making them easier to segment accurately. In contrast, the subtler features of unripe strawberries may have presented greater segmentation challenges. It is also possible that dataset bias could have played a role, with the model potentially being exposed to more ripe strawberries or synthetic images that emphasized more pronounced or varied features of the ripe class, leading to better performance in that category.

One challenge associated with synthetic dataset generation is the presence of minor artifacts, such as unnatural leaf intersections, uniform lighting conditions, and occasional texture mismatches. While procedural modeling techniques were employed to randomize plant structures, some level of artifact generation is inevitable in Blender-based datasets. These effects were mitigated through domain adaptation, however, as the Deep Domain Confusion (DDC) technique encouraged the model to focus on robust feature representations rather than dataset-specific artifacts. The results of this study suggest that, despite these imperfections, the model generalizes well to real-world images.

### 4.1 Generalizability to other agricultural contexts

The methodology presented in this study is not limited to strawberry segmentation but can be generalized to other agricultural applications. The synthetic data generation pipeline in Blender can be adapted for different fruit types by modifying object textures, colors, and shapes. Additionally, the SwinUNet segmentation framework, combined with Deep Domain Confusion (DDC), is applicable to other controlled-environment and open-field agriculture scenarios. While additional real-world calibration would be necessary for outdoor conditions, the proposed approach offers a scalable and cost-effective way to expand deep learning applications in precision agriculture.

### 4.2 Adaptability of SwinUNet and DDC to other applications

The SwinUNet architecture and DDC framework are highly adaptable beyond strawberry segmentation. Given SwinUNet’s self-attention mechanisms, it can be trained on datasets for other fruits (e.g., apples, tomatoes, grapes) with minimal adjustments. Similarly, DDC can help bridge synthetic-to-real transitions in open-field agriculture where shadows, variable lighting, and diverse backgrounds introduce new challenges. Future work could further explore multimodal fusion (e.g., combining RGB with NIR imaging) to enhance segmentation performance under real-world agricultural conditions.

## 5. Future work

Looking ahead, there are several promising avenues for enhancing the functionality and accuracy of this image processing system. First, there is potential for improvement in the quality of the generated images to make them more closely resemble real images. For example, the Blender model could be further improved by preventing the intersection of different plant components, such as leaves bisecting other leaves or stems piercing through strawberries. Additionally, adding greater variation to properties such as leaf and stem shading, as well as diversifying the shapes of leaves and strawberries, could further refine the model. Improving the photorealism of the strawberry itself is another potential avenue for refinement. In addition, incorporating different backgrounds and environments could enhance the diversity of the dataset, potentially improving the model’s ability to generalize across various real-world scenarios. These enhancements would likely lead to better model training outcomes and more accurate segmentation in practical applications.

Second, in the future, the adoption of stereoscopic cameras [50] for capturing real-time images could significantly advance our capabilities. By utilizing such technology, it would be possible to accurately measure the size and volume of objects, such as strawberries. This dimensional data could provide valuable additional information to determine the optimal timing for harvesting.

Future work will also explore GAN-based refinement of synthetic datasets to further reduce potential artifacts. Additionally, future work could incorporate multimodal fusion (as in U3M [13]) to further improve segmentation robustness in diverse environmental conditions.

Furthermore, in real-world farm environments, sensor noise, uncontrolled lighting variations, and physical occlusions from leaves or other fruits pose significant challenges to segmentation accuracy. The model also lacks the ability to handle physically damaged strawberries, where fruit textures may be significantly different from synthetic or healthy training samples. These factors indicate a need for on-site domain adaptation techniques and real-time calibration of segmentation parameters to ensure optimal performance in uncontrolled agricultural settings.

Additionally, exploring various Transformer models could further enrich our understanding of their effectiveness in segmentation tasks. Experimenting with models like the SETR [51] could provide insightful comparisons with the currently employed algorithms, potentially revealing strengths or weaknesses that could inform future improvements and adaptations in our approach.

Also, future research could explore the effects of training with zoomed-in images to determine if synthesizing images from a close distance influences accuracy.

## 6. Summary & conclusions

This study demonstrates the successful application of Blender-generated synthetic data for training a Vision Transformer model that accurately differentiates between ripe and unripe strawberries, achieving a Dice Similarity Coefficient above 90%. While these results highlight the model’s effectiveness for fruit ripeness detection, they also reveal the limitations of domain adaption techniques like Deep Domain Confusion, which cannot fully bridge the gap between synthetic and real-world data. However, increasing the dataset size has proven to mitigate some of these limitations, as larger datasets lead to improved segmentation outcomes.

By generating large and diverse synthetic datasets with Blender, this approach offers significant time and cost savings compared to traditional resource-intensive data collection methods. The study demonstrates how Blender-generated datasets can be customized to specific environments and conditions. Ultimately, this research highlights the potential of synthetic datasets as a cost-effective and efficient solution for addressing data scarcity in agricultural applications.
